# Risk factors analysis and prediction model construction of LRTI in head and neck cancer patients with tracheostomy based on subglottic sputum aspiration volume

**DOI:** 10.3389/froh.2026.1771262

**Published:** 2026-03-20

**Authors:** Jie Zhang, Wei Deng, Zhentao Lao, Yudong Xiao, Yinyan Chen, Guiqing Liao, Le Yang, Yujie Liang

**Affiliations:** 1 Department of Oral and Maxillofacial Surgery, Hospital of Stomatology, Guanghua School of Stomatology, Sun Yat-sen University, Guangzhou, Guangdong, China; 2 Guangdong Provincial Key Laboratory of Stomatology, Sun Yat-sen University, Guangzhou, Guangdong, China

**Keywords:** head and neck cancer, lower respiratory tract infection, nomogram, subglottic sputum aspiration volume, tracheotomy

## Abstract

**Objective:**

Most head and neck cancer (HNC) patients had postoperative aspiration and even lower respiratory tract infections (LRTI). This study aimed to investigate the association between subglottic sputum aspiration volume (SSAV) and the onset of LRTI in HNC patients with tracheostomy. We further sought to identify independent risk factors and construct a predictive model for postoperative LRTI in this patient population.

**Methods:**

This study retrospectively enrolled 235 HNC patients with intraoperative tracheotomy from June 2018 to November 2022. Subglottic sputum aspiration volume (SSAV) and other clinical data were collected. Univariate and multivariable analyses were performed to construct a logistic regression model. According to the model, a Nomogram was created to visualize the risk of LRTI, and another 66 patients from March 2023 to May 2023 were recruited to validate the prediction model.

**Results:**

The univariate analysis showed that preoperative head and neck surgery history, WBC, PCT, CRP, tumor T stage, tumor N stage, and the SSAV changes had significantly positive relationships with postoperative LRTI. PCT, CRP, tumor T stage, SSAV Range, SSAV Max, and SSAV Min were demonstrated to be independent risk factors. Pathogen analysis revealed that the microbiota of the lower respiratory tract infection was *Pseudomonas aeruginosa*, *Staphylococcus aureus*, and *Acinetobacter baumannii* complex group. Model validation analysis showed that the model fit well with the actual situation (AUC = 87.9%, 95%CI:0.767–0.992).

**Conclusion:**

SSAV is an unneglectable and meaningful clinical parameter, and the changes in SSAV can predict the risk of LRTI in patients with intraoperative tracheotomy. A new prediction model is satisfactory in predicting LRTI after intraoperative tracheotomy.

## 1 Introduction

Head and neck cancer (HNC) is one of the seven most common cancers in the world, with considerable morbidity and mortality (1, 2). Surgery remains a primary treatment but can induce severe life-threatening complications such as airway obstruction and laryngeal spasm due to severe postoperative swelling and secretion accumulation (1, 3–6). Tracheotomy can efficiently prevent airway obstruction, significantly reduce the risk of postoperative critical illness, and shorten the time in the intensive care unit (7, 8). However, this procedure disrupts airway integrity and, with long-term catheterization, facilitates colonization of the lower respiratory tract by opportunistic pathogens, thereby increasing the risk of postoperative lower respiratory tract infection (LRTI) (9, 10).

A key mechanism predisposing to LRTI is the aspiration of oropharyngeal secretions past the tracheal tube cuff. The subglottic space above the cuff can accumulate secretions from surgical wounds, mucosa, and saliva. While continuous or intermittent suctioning of this space, measured as the subglottic sputum aspiration volume (SSAV), is a routine nursing practice to reduce aspiration risk (11). However, when cuff displacement, low air pressure, or improper nursing practices occurs, it is inevitable that some secretions may leak or be aspirated into the lower respiratory tract, which may manifest as symptoms including coughing, expectoration, fever, and potentially lead to lower respiratory tract infections (12). Despite numerous studies on the accumulated secretions in the oropharynx and LRTI after head and neck surgery, there have been few studies on the quantitative analysis of the correlation between them. As we observed in clinical practice, it presented as an abrupt and considerable decline or marked sudden increase in subglottal suction volume. It aroused our interest in the abrupt disappearance or emergence of SSAV, could be responsible for the onset of the LRTI. In this study, we proposed that SSAV dynamics serves as a predictive indicator for LRTI among HNC patients with tracheostomy. We aimed to explore the risk factors of postoperative LRTI and constructed a prediction model to predict the potential risk of LRTI in HNC patients with intraoperative tracheotomy.

## 2 Methods

### 2.1 Study design

This study retrospectively analyzed the risk factors of LRTI among HNC patients with tracheostomy based on SSAV. And we established a logistic regression model, with drawing a Nomogram on this basis. At last, we used a quasi-external validation set to verify the efficiency of the prediction model.

### 2.2 Patients

The patient data set consisted of two parts: retrospective study and model validation. In the retrospective study, the recruited subjects were consecutive patients diagnosed with HNC from June 2018 to November 2022 in the Hospital of Stomatology, Sun Yat-Sen University. All patients had undergone surgery and intraoperative tracheotomy (the patient inclusion flowchart as shown in Supplementary Figure S1). Tracheotomy was performed based on standardized clinical indications, including one or more of the following: a) Anticipated postoperative upper airway obstruction due to postoperative edema; b) Planned major reconstruction with free flaps requiring prolonged mechanical ventilation; c) History of prior radiotherapy to the head and neck region with associated tissue fibrosis and reduced airway compliance. Tumor subsites were classified according to the International Classification of Diseases for Oncology (ICD-O-3) into the following anatomical categories: oral cavity (including tongue, floor of mouth, buccal mucosa, hard palate, alveolar ridge), oropharynx, hypopharynx, larynx, salivary glands, and others. Primary tumor location was documented from surgical and histopathological reports. Patients were excluded when a) they were diagnosed with LRTI before hospitalization, b) they refused to participate or decided to withdraw from the study, and c) the clinical data were insufficient. In the model validation period, we included consecutive subjects from March 2023 to May 2023. The inclusion and exclusion criteria are the same as before. Finally, a total of 235 patients with HNC after intraoperative tracheotomy were included in the retrospective study, and 66 cases were included in the validation set.

### 2.3 The collection of clinical data

Clinical data pertaining to all patients were collected, including the basic information, comorbidity, clinical and pathological diagnoses, hospital course, surgery procedures, and complications. Serum levels of inflammatory biomarkers (CRP and PCT) were ascertained at timepoints proximal to key clinical events. For patients in the LRTI group, measurements were taken on the day preceding of infection diagnosis. For patients in the non-LRTI group, measurements were taken on the day preceding or the day of planned tracheal tube removal. These pre-event values, obtained before the respective clinical endpoints, were used in the model to evaluate the association between the immediate pre-outcome inflammatory status and the risk of LRTI.

SSAV was defined and recorded as the daily total volume of secretions aspirated from above the endotracheal tube cuff from 8:00 to 8:00 the following day, until tracheal tube removal. Aspiration was performed every 2 h by trained nursing staff using a standard syringe connected to the subglottic suction port. The volume was read directly from the syringe scale and recorded. To ensure accuracy, oral and pharyngeal secretions were cleared prior to each subglottic aspiration. All nursing staff followed the same standardized protocol. Diagnosis of LRTI was based on a composite of clinical, radiological, and microbiological criteria adapted from ERS/ESCMID guidelines (13, 14). Specifically, patients were considered to have LRTI if they presented with: a) Clinical signs: new/worsening cough, sputum, dyspnea, fever (>38.0 °C), AND b) Radiological evidence: new/progressive infiltrates on chest imaging, AND/OR c) Microbiological confirmation: pathogenic bacteria cultured from lower respiratory tract specimens with compatible symptoms. All cases were reviewed independently by two clinicians to confirm adherence to these criteria. In the analysis of SSAV, we mainly focused on the values and their variations. Specifically, we evaluated the maximum SSAV (SSAV max) in conjunction with the range between maximum and minimum values (SSAV Range) to clarify the impact of suction volume itself on LRTI. Furthermore, we analyzed daily variations of SSAV to ascertain their impact on LRTI, encompassing the maximum increases (SSAV Increase), maximum decreases (SSAV Decrease), and the overall range of these fluctuations (SSAV Variation). Specifically, we defined the daily change (Δ*V_d_*) as the difference between the SSAV of day d(*V_d_*) and the preceding day d(*V_d_*_−1_). SSAV Increase = max (*V_d_* − *V_d_*_−1_), where (*V_d_* − *V_d_*_−1_) > 0; SSAV Decrease = max (*V_d_* − *V_d_*_−1_), where (*V_d_* − *V_d_*_−1_) < 0; SSAV Variation = (SSAV Increase) + (SSAV Decrease). With respect to temporal considerations, for the LRTI group, we documented SSAV from the time of surgery until the clinical diagnosis of infection; in contrast, for the non-LRTI group, we recorded changes from surgery until balloon catheter removal (or endotracheal tube replacement).

### 2.4 Statistical analysis

The clinical data were analyzed with SPSS (v25.0) and R (v4.2.2). Prior to analysis, the extent of missing data was assessed for all candidate variables. In this study, given the low proportion of missingness and to maintain consistency across analyses, cases with any missing data in the variables of interest were excluded from the model development and validation analyses. In univariate analysis, we compared categorical variables by Fisher's exact test and *χ*^2^ test and continuous variables with a *t*-test or Mann–Whitney *U-*test where appropriate. To complement the analysis using continuous variables and to facilitate clinical interpretation, key SSAV parameters were also transformed into binary clinical categories. The optimal thresholds for this categorization were derived empirically using receiver operating characteristic (ROC) curve analysis to maximize the ability to discriminate between patients with and without LRTI. Specifically, the cut-off value for each variable was selected to maximize Youden's index (J = sensitivity + specificity − 1). Patients were then categorized as “high” or “low” risk based on whether their value exceeded these diagnostic performance-optimized thresholds. In multivariable analysis, we set LRTI as the outcome index and the days of LRTI or trachea catheterization without LRTI as the time variables. The logistic regression model was used to detect independent risk factors for LRTI. Candidate predictors for the multivariate logistic regression model were initially selected based on clinical relevance, existing literature, and results from univariate analyses (variables with a univariate association of *P* < 0.05 with LRTI were initially considered to avoid prematurely excluding potential predictors). Potential multicollinearity among the selected variables was assessed using Variance Inflation Factors (VIFs) to indicate no severe multicollinearity. Additionally, the estimate of the association was expressed as an odds ratio (OR) with corresponding confidence intervals of 95%, and the two-tailed significance level of 0.05 was used. The predictive accuracy of the final logistic regression model was evaluated by assessing its discrimination and calibration. The area under the receiver operating characteristic curve (AUC) with 95% confidence interval was used to measure the discrimination. Calibration was assessed via calibration plots and the Hosmer-Lemeshow goodness-of-fit test. These performance metrics were reported for both the model development cohort and the validation set.

## 3 Results

### 3.1 The baseline of patients' characteristics

A total of 235 patients were included in the retrospective study. They were divided into the LRTI group and a non-LRTI group according to whether LRTI occurred after intraoperative tracheotomy. There were 60 in the LRTI group and 175 in the non-LRTI group, with the ratio of male to female being 72.3% (80.0% in the LRTI group and 69.7% in the non-LRTI group) and 27.7% (20.0% in the LRTI group and 30.3% in non-LRTI group). The mean age was 52 years old in the LRTI group and 50 years old in the non-LRTI group. There was no significant difference in gender and age. The basic conditions of patients with a history of smoking, drinking, and chewing betel nuts are shown in Table 1. There was no statistically significant difference in the preoperative history of pneumonia and preoperative history of radiotherapy or chemotherapy between the two groups. The mean time of extubation in the LRTI group was 6.81 days, which was significantly longer than that in the non-LRTI group (3.95 days, *P* < 0.001).

**Table 1:** Patients' demographic and baseline characteristics.

	LRTI group	Non-LRTI group	*P* value
	(*n* = 60)	(*n* = 175)	
Gender			0.124
Male	48 (80.00%)	122 (69.71%)	
Female	12 (20.00%)	53 (30.29%)	
Age (year)^a^	52 ± 11	50 ± 10	0.791
Smoking history			0.583
Yes	34 (56.67%)	92 (52.57%)	
No	26 (43.33%)	83 (47.43%)	
Alcohol consumption			0.967
Yes	18 (30.00%)	53 (30.29%)	
No	42 (70.00%)	122 (69.71%)	
Chewing betel nut			0.775
Yes	9 (15.00%)	29 (16.57%)	
No	51 (85.00%)	146 (83.43%)	
Diabetes			0.732
Yes	4 (6.67%)	15 (8.57%)	
No	56 (93.33%)	160 (91.43%)	
Pre-operative HNSH			0.854
Yes	7 (11.67%)	22 (12.57%)	
No	53 (88.33%)	153 (87.43%)	
Pre-operative RTH			0.091
Yes	0 (0)	8 (4.57%)	
No	60 (100.00%)	167 (95.43%)	
Pre-operative CTH			0.282
Yes	1 (1.67%)	8 (4.57%)	
No	59 (98.33%)	167 (95.43%)	
Days of intubation^a^	6.81 ± 3.26	3.95 ± 1.54	**<0** **.** **001** ***

^a^ Mean ± standard deviation.

*** *P* value less than 0.001.

HNSH, head and neck surgery history; RTH, radiotherapy history; CTH, chemotherapy history.

The bold values represents the significant statistical differences.

We further analyzed the LRTI group patients and found that the tumor occurred mainly in the tongue, followed by the floor of the mouth and cheek (as shown in Supplementary Table S1). Squamous cell carcinoma (SCC) was the main pathological pattern of HNC, and tumor size was focused on T3–4. To get insight into the influence of surgical methods on LRTI, we calculated the basic surgical options of patients in the LRTI group (as shown in Supplementary Table S2). Significantly, the proportion of patients with mandibular incision or segmental resection involved in the surgical plan reached up to 60.0%, and 28.3% of patients had masticatory muscle defects, all of which may affect postoperative swallowing function, wound recovery, and airway continuity, increasing the risk of LRTI after intraoperative tracheotomy. Unfortunately, it did not show a statistically significant difference in our data.

For the patients in the LRTI group, we also carried out an etiological examination of the upper airway secretions and the subglottic sputum. The results of the bacterial culture are shown in Supplementary Table S3. LRTI patients mostly experienced multiple bacterial infections and multiple drug resistance. The main pathogens detected in the LRTI patients were *Klebsiella pneumoniae*, followed by *Pseudomonas aeruginosa*, *Staphylococcus aureus*, and *Acinetobacter baumannii* complex group. Multiple drug-resistant pathogens infection accounted for a high proportion 46.0%, and non-drug resistant pathogens infection accounted for just 18.9%, suggesting that the pathogenic conditions of LRTI patients with HNC after intraoperative tracheotomy are complex and diverse. The emergence of this situation has brought great difficulty in clinical diagnosis and treatment.

### 3.2 Risk factors of LRTI in HNC patients with intraoperative tracheotomy

We included factors that may be related to LRTI for univariate analysis. The results are shown in Table 2. WBC(*P* < 0.001), PCT (*P* < 0.001), and CRP (*P* < 0.001), those generally recognized as indicators of infection, showed significant statistical differences between the two groups. Opposite to our expectations, the Body Mass Index (BMI) (*P* = 0.554) and diabetes (*P* = 0.847) may not be independent risk factors for LRTI. In terms of tumor-related parameters, the tumor T stage in the LRTI group was significantly higher than in the non-LRTI group (*P* < 0.001). As for the tumor N stage, a higher proportion of patients with local lymph node metastasis was observed in the LRTI group; nevertheless, this difference did not achieve statistical significance.

**Table 2:** The univariate and multivariate analysis of risk factors of post-operative LRTI in the HNC patients with intraoperative tracheotomy.

Characteristics	LRTI	non-LRTI	Univariate analysis	Multivariate analysis	
	(*n* = 60)	(*n* = 175)	*P* value	*P* value	OR (95%CI)
BMI^a^	22.31 ± 3.26	22.61 ± 3.43	0.554		
Diabetes			0.847		
Yes	4 (6.67%)	15 (8.57%)			
No	56 (93.33%)	160 (91.43%)			
Chewing betel nut			0.775		
Yes	9 (15.00%)	29 (16.57%)			
No	51 (85.00%)	146 (83.43%)			
Smoking history			0.217		
Yes	34 (56.67%)	83 (47.43%)			
No	26 (43.33%)	92 (52.57%)			
Alcohol consumption			0.967		
Yes	18 (30.00%)	53 (30.29%)			
No	42 (70.00%)	122 (69.71%)			
WBC^a^	12.76 ± 5.18	9.45 ± 2.95	<0.001***		
PCT^a^	0.79 ± 1.58	0.22 ± 0.17	0.007**	0.071	5.643 (0.862,36.964)
CRP^a^	92.18 ± 53.39	30.90 ± 28.46	<0.001***	<0.001***	1.029 (1.016,1.042)
Tumor T stage^b^			<0.001***		
≤2	4 (7.69%)	49 (35.77%)			Reference
3	20 (38.46%)	48 (35.04%)		0.013*	6.963 (1.510,32.111)
4	28 (53.85)	40 (29.20%)		0.014*	6.446 (1.459,28.477)
Tumor N stage^b^			0.081		
0	23 (44.23%)	80 (58.39%)			
≥1	29 (55.77%)	57 (41.61%)			
Tumor sites			0.448		
Tongue	24 (40.00%)	68 (38.86%)			
The floor of the mouth	14 (23.33%)	27 (15.43%)			
Cheek	6 (10.00%)	26 (14.86%)			
Others	16 (26.67%)	54 (30.85%)			
Pathological pattern			0.082		
SCC	55 (91.67%)	144 (82.29%)			
Non-SCC	5 (8.33%)	31 (17.71%)			
SSAV					
Max^a^	46.31 ± 25.44	28.41 ± 19.19	<0.001**		
Range^a^	32.28 ± 22.02	20.48 ± 15.61	0.001**	0.042*	1.047 (1.002,1.095)
Increase^a^	23.47 ± 19.24	8.16 ± 13.61	<.001**	0.074	1.029 (0.997,1.063)
Decrease^a^	26.02 ± 18.58	12.67 ± 11.94	<0.001**	0.030*	1.059 (1.006,1.115)
Variation^a^	29.37 ± 19.75	16.47 ± 12.36	<0.001***		

^a^ Mean ± standard deviation.

^b^ Excluding 46 cases of jawbone tumors (8 cases in LRTI group and 38 cases in non-LRTI group), including ameloblastoma, squamous cell carcinoma and osteosarcoma of the jaw.

* *P* value less than 0.05.

** *P* value less than 0.01.

*** *P* value less than 0.001.

OR, odds ratio; 95%CI, 95% confidence interval; BMI, body mass index; WBC, white blood cell; PCT, procalcitonin; CRP, C-reactive protein; SCC, squamous cell carcinoma; SSAV, Subglottic sputum aspiration volume.

The volume of endotracheal suctioning in postoperative patients frequently exhibits dynamic variability. As the surgery wound heals and the patient's immune status improves, it generally demonstrates a progressive decline until normal swallowing function is reinstated. However, in instances of leakage, aspiration, or respiratory infection, the suctioning volume may undergo abrupt increases or decreases. In our research, the results of the univariate analysis indicated that both the SSAV Max (*P* < 0.001) and the SSAV Range (*P* = 0.001) were significantly greater in the LRTI group compared to the non-LRTI group. Furthermore, we transformed these continuous variables into ordered categorical variables based on the diagnostic curve, and the newly defined variables continued to demonstrate statistical significance, suggesting an increased risk of LRTI when the endotracheal suction volume exceeds 46.31 mL or when its range surpasses 32.28 mL. Notably, both groups exhibited significant differences regarding increments in SSAV Increase, SSAV Decrease, and SSAV Variation. Specifically, a higher incidence of LRTI is associated with sudden increases exceeding 23.47 mL or decreases surpassing 26.02 mL in SSAV.

### 3.3 Multi-factor analysis, prediction model construction and validation

**Figure 1:**
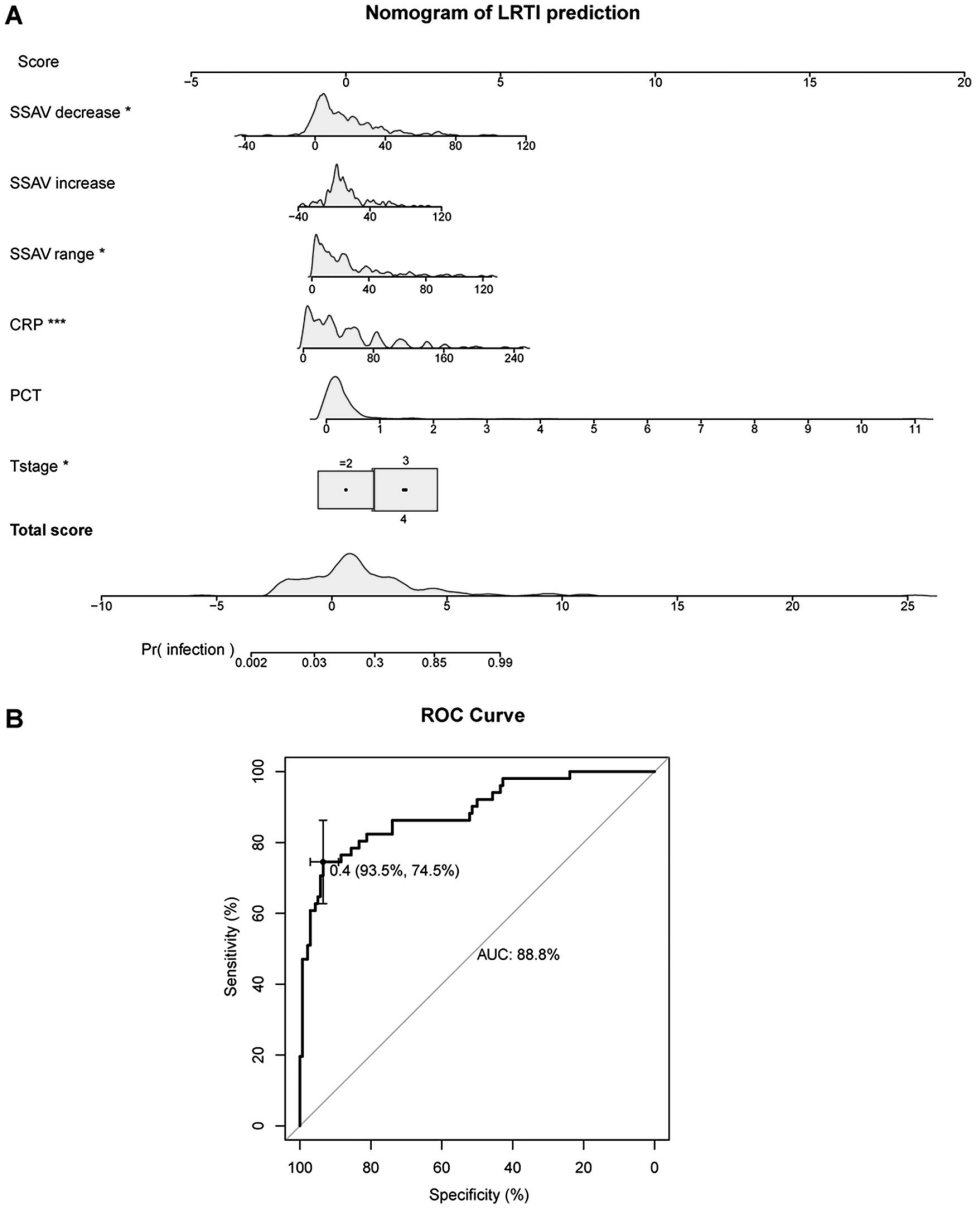
Nomogram of logistic model predicts the occurrence of LRTl in HNC patients with tracheotomy. **(A)** Nomogram of LRTI prediction. Scores are assigned based on SSAV decrease, SSAV increase, SSAV range, CRP, PCT, and T stage by drawing a vertical line upward from the corresponding values to the “Scores” line. The sum of these scores, plotted on the “Total scores” line, corresponds to prediction of LRTI rate plotted on the “Pr(infection)” line. **(B)** Receiver Operating Characteristics (ROC) curve of the nomogram. AUC, area under the curve.

Variables identified through the integrated selection process (univariate screening *P* < 0.05, combined with clinical relevance and absence of multicollinearity, VIFs provided in Supplementtary Table S4) were included in the final multivariate logistic regression model (as shown in Table 2). In the model, the Hosmer and Lemeshow Test showed the *P*-value (*P* = 0.328) greater than the significance level. The area under the diagnostic curve tested to be 0.868, indicating that the model demonstrates a good fit. According to the established logistic regression model, a visual charts-nomogram was drawn to predict the risk of LRTI in HSC patients after intraoperative tracheotomy (as shown in Figure 1A). The C index of the model is 0.888, and the calibration plot showed a close fit between the predicted probability and the actual observed frequency of LRTI (as shown in Figure 1B and Supplementary Figure S2A), indicating satisfactory efficiency in predicting LRTI after intraoperative tracheotomy. In the quasi-external validation data set, we obtained 66 subjects and utilized our model to estimate the probability of LRTI occurrence. The demographic characteristics of the quasi-external validation set were summarized in the Supplementary Table S5. By comparing the LRTI patients predicted by the model with the actual LRTI patients, we obtained that the prediction accuracy of the model was 0.648, the sensitivity was 0.636. Furthermore, the AUC of the diagnostic curve in the validation set tested to be 0.879 (95%CI:0.767–0.992), showing that the model had a good fit with the actual situation (as shown in Supplementary Figure S2B). The clinical utility of the nomogram was further evaluated using Decision Curve Analysis (DCA) in the validation set (Supplementary Figure S2C).

## 4 Discussion

Our work explored the clinical risk factors of postoperative LRTI in HNC patients with tracheostomy. Using univariate and multivariate analysis, we identified several preoperative and perioperative factors correlated with postoperative LRTI. In contrast to prior studies that primarily focused on static measures of aspiration or infection biomarkers, our study emphasizes the prognostic value of monitoring dynamic changes in SSAV, which reflects real-time secretion management and airway competence in tracheostomized patients (15, 16). We established a predictive model for LRTI based on variations mainly in SSAV, which exhibited strong performance during both internal and external validation. The application of this model facilitates clinicians in assessing the risk of LRTI in a systematic and efficient manner, thereby providing a robust theoretical framework for physicians to develop preventive interventions against LRTI.

Through quantitative evaluation of SSAV, our study identified that a sudden reduction exceeding 46.31 mL or when its range surpasses 32.28 mL in SSAV over two consecutive days may be an independent risk factor for postoperative LRTI. Typically, as the wound heals and swallowing function improves, subglottal suction volume generally decreases steadily (17). Thus, what happened to the suddenly reduced SSAV, and where had they gone? A plausible explanation is that insufficient adherence between the cuff and the airway folds may result in leakage of subglottal secretions (18, 19). Recent studies have further elucidated that cuff pressure fluctuations and subglottic secretion clearance are critical in preventing aspiration, yet most prior works did not quantify secretion volume trends as a predictive tool (20, 21). However, due to individual variations in airway anatomy, such leakage caused by inadequate adhesion between the cuff and the airway folds is often unpredictable. Previous research indicates that alterations in cuff pressure may result in the leakage of secretions above the cuff, thereby contributing to the onset of LRTI (22). Factors such as patient positioning, the integrity of the cuff device, and any procedural nuances during operations by doctors and nurses can all influence changes in cuff pressure (23). In the study conducted by Carter, E. L. and A. Duguid, the leakage rate of upper secretions was found to be 2.25 ± 1.49 mL/min with no monitoring of cuff pressure, and a significant increase in the risk of LRTI was observed when the cuff airway pressure fell below 20 mmHg (22). Li, B. G.'s research emphasized continuous airway pressure monitoring can reduce the occurrence of leakage and significantly reduce the incidence of lower respiratory infections (19). However, excessive cuff airway pressure may also lead to adverse effects, such as tracheal edema (24). Recent evidence supports that automated cuff pressure control systems may reduce aspiration risk, yet their impact on SSAV trends remains underexplored (21, 25). Studies have shown that continuous subglottal suction after tracheostomy can effectively prevent LRTI (26, 27). It is worth noting that suctioning frequency is not necessarily better the more frequent it is, as excessive suctioning frequency may also cause damage to the airway, leading to increased SSAV fluctuations and higher risks of leakage and aspiration (28). Additionally, during the suctioning process, medical procedures may also cause other iatrogenic injuries, which may lead to the occurrence of LRTI (28). The decline in swallowing function after HNC surgery often results in the accumulation of residues in the oropharynx and oropharynx, which cannot be effectively cleared by swallowing action (29). This leads to an increase in the accumulation of residues below the vocal cords, further increasing the risk of leakage and aspiration, and also increasing the possibility of LRTI (29, 30). Unlike previous studies that relied on subjective swallowing assessments, we designed our study to utilize SSAV as a continuous, quantitative surrogate marker for swallowing competence. While acknowledging that swallowing dysfunction is the fundamental etiology of aspiration, instrumental evaluations, such as Flexible Endoscopic Evaluation Swallowing (FEES), typically offer only intermittent snapshots of function. In contrast, SSAV dynamics reflect the cumulative consequence of swallowing impairment over time. Therefore, our objective SSAV metrics bridge the gap between functional evaluation and infection outcomes by providing a real-time alert system for aspiration risk. There have been reports indicating that the incidence of aspiration among tracheotomy patients ranges from 50% to 87% (31–34). In Denys Loeffelbein et al.'s research, 18.8% of the 648 patients experienced pulmonary complications after undergoing oral and maxillofacial surgery combined with tracheotomy (35). The incidence of LRTI after tracheotomy in HNC patients reported by Schuderer JG et al. is nearly the same, at 15.4% (36). Current research indicates that this may be associated with alterations in the swallowing mechanism following tracheostomy, which may increase the risk of aspiration (37, 38). Reflected in the SSAV index, it's where an increase in the amplitude of observable SSAV fluctuations can be noted. This result can be further verified in the Modified Evans Blue Dye Test (MEBDT) and FEES studies in HNC patients with tracheotomy (39, 40). Compared with MEBDT and FEES, the monitoring data of SSAV is more convenient and accessible. However, the efficiency and benefits of SSAV and MEBDT/FEES in the prevention and management of LRTI still need further research.

Within the realm of SSAV research, it is crucial to not only investigate changes in its quantity but also to consider the intrinsic properties and composition of subglottal accumulations, as these elements are fundamentally responsible for the onset of LRTI. The composition of subglottic accumulation primarily consists of secretions from surgical incisions, salivary glands, and mucosal membranes, as well as airway secretions and foreign substances that may inadvertently enter the subglottis during the processes of swallowing and drinking (41, 42). In comparison to other regions of the body, the oral cavity serves as a site rich in bacteria, characterized by a warm and moist environment that maintains a unique dynamic equilibrium of microbial ecology (43, 44). However, head and neck surgery trauma promotes the colonization of pathogenic microorganisms originating from the oral cavity and nasal passages (44). When aspiration occurs, these microorganisms can invade the lower respiratory tract, disrupting microbial balance and ultimately contributing to the onset of LRTI. This condition further elevates the risk of infection by multi-drug-resistant bacterial strains (44–48). According to previous reports, the pathogenic bacteria that cause LRTI in patients after tracheostomy were mainly Gram-negative bacteria such as *Pseudomonas aeruginosa*, *Klebsiella pneumoniae*, and *Acinetobacter baumannii* (49). In a study involving HNC patients with postoperative LRTI following tracheostomy, it was observed that individuals without aspiration exhibited a lower respiratory microbiome more closely resembling the nasal microbiome. In contrast, those with aspiration demonstrated a lower respiratory microbiome that aligned more closely with the gastrointestinal microbiome, resulting in a hybridization of upper respiratory and gastrointestinal microbial communities (50, 51). Recent metagenomic studies have further characterized this dysbiosis in tracheostomized patients, supporting our findings of polymicrobial infections (52, 53). In our study, the most predominant pathogens identified were *Klebsiella pneumoniae*, followed by *Pseudomonas aeruginosa*, *Staphylococcus aureus*, and *Acinetobacter baumannii* complex group. This finding resembles the microbial mixture observed in the upper respiratory and upper gastrointestinal tracts, further substantiating that a decline in swallowing function following tracheostomy elevates the risk of aspiration and ultimately contributes to the onset of LRTI. Moreover, it also reminds us that in clinical practice, more attention should be paid to swallowing function rehabilitation for HNC patients after tracheostomy who exhibit significant fluctuations in SSAV. Additionally, it is essential to ensure postoperative proper oral hygiene and to conduct early sampling of pathogenic microorganisms for timely detection and intervention, which can facilitate the prevention and management of postoperative infections.

In the analysis of the patient's general condition, preoperative radiotherapy is recognized as a significant risk factor for LRTI following intraoperative tracheotomy (54). Radiotherapy can induce submucosal fibrosis, which subsequently impacts neck mobility and leads to anatomical and physiological alterations in the pharyngeal airway, thereby exacerbating the risk of LRTI during the perioperative period (55, 56). Patients with HNC undergoing tracheotomy often require prolonged surgical procedures and anesthesia. Typically, these cases encompass intricate procedures such as vascularized flap transplantation and microvascular reconstruction (35, 36, 57). Notably, although patients undergoing these surgeries often present as smokers, heavy drinkers, or elderly individuals with compromised health conditions, our study still suggests that age, sex, diabetes status, and other general health factors may not function as reliable predictors of LRTI as previously anticipated. This contrasts with some earlier models that emphasized comorbidities; our data suggest that in surgically managed HNC, procedural and secretion-related factors may outweigh traditional risk profiles (58, 59). Furthermore, it is noteworthy that the proportion of surgical protocols involving the mandible reaches up to 60% in the LRTI group. Mandibular defects and reconstructions are closely linked to the extent of surgical wounds and alterations in airway structure. This suggests that airway protection strategies and recovery of physiological functions—often overlooked in clinical practice—may significantly elevate the risk of postoperative LRTI in such patients.

The prediction model developed in this study translates identified risk factors into direct clinical utility. The derived nomogram quantifies individual risk by integrating readily available pre-extubation variables, including SSAV dynamics, CRP, PCT, and T stage. This facilitates risk stratification prior to surgery and extubation, enabling clinicians to target high-risk patients for interventions such as intensified monitoring, prolonged airway protection, or enhanced pulmonary hygiene. Furthermore, it shifts the paradigm of SSAV monitoring from a passive nursing metric to an active component of dynamic risk assessment, where acute changes can function as real-time clinical alerts. While promising for personalized management and resource optimization, the model's application is subject to several limitations. First, our study design may introduce inherent biases, including selection bias and unmeasured confounding. Although we employed consecutive enrollment and multivariable adjustment, residual confounding from unrecorded or unavailable clinical variables. Second, regarding swallowing function, we did not incorporate gold standard swallowing function evaluations into the analytical framework. This was a deliberate consideration in our study design, which aimed to identify accessible bedside predictors rather than to characterize mechanistic etiologies. We acknowledge that SSAV functions clinically as a downstream surrogate for swallowing impairment rather than a mechanistically distinct factor. However, our findings demonstrate that this surrogate holds unique independent predictive value. Unlike functional tests that require specialized equipment and scheduling, SSAV acts as a continuous monitor, capturing the real-world fluctuation of secretion management that directly precedes infection. Third, in the validation stage of our prediction model, there is a lack of indicators to verify the clinical benefits of interventions on outcomes. Finally, our study is limited by the absence of a true external validation cohort, which restricts the generalizability of the findings. Future prospective studies, incorporating standardized swallowing evaluations and protocolized data collection, are needed to validate and refine this model while minimizing these biases.

## 5 Conclusions

In summary, our study identifies SSAV dynamics as a critical predictor of LRTI in HNC patients with tracheotomy. Our predictive model, integrating SSAV and clinical factors, offers a robust tool for risk stratification, enabling early intervention for high-risk patients. Future prospective, multi-center validation is essential to confirm its clinical utility and generalizability.

## Data Availability

The original contributions presented in the study are included in the article/Supplementary Material, further inquiries can be directed to the corresponding authors.
