# Supplementary material for: Risk factors analysis and prediction model construction of LRTI in head and neck cancer patients with tracheostomy based on subglottic sputum aspiration volume
Source: Front Oral Health. 2026 Mar 20;7:1771262. doi: 10.3389/froh.2026.1771262 (PMC13047123; doi:10.3389/froh.2026.1771262)
Supplement: Supplementary file 1 [file Datasheet1.pdf]

Supplementary Material

1 Supplementary Tables

Supplementary Table 1 Tumor characters of LRTI group patients.

Characteristics	LRTI group n=60	Non-LRTI group n=175	P value
**Tumor sites**			0.524
Tongue	24 (40.00%)	68 (38.9%)	
The floor of the mouth	14 (23.33%)	27 (15.4%)	
Buccal mucosa	6 (10.00%)	26 (14.9%)	
The lower gingiva and alveolar ridge	11 (18.33%)	37 (21.1%)	
Others	5 (8.33%)	17 (9.7%)	
**Pathological pattern**			0.082
SCC	55 (91.67%)	144 (82.3%)	
non-SCC	5 (8.33%)	31 (17.7%)	
**Tumor T stage** [b]			< 0.001***
2	4 (7.69%)	49 (35.77%)	
3	20 (38.46%)	48 (35.04%)	
4	28 (53.85%)	40 (29.19%)	
**Tumor N stage** [b]			0.075
0	23 (44.23%)	80 (45.7%)	
1	10 (19.23%)	16 (9.1%)	
2	9 (17.31%)	29 (16.6%)	
3	10 (19.23%)	12 (6.9%)	

Note: [b] excluding 46 cases of jawbone tumors (8 cases in LRTI group and 38 cases in non-LRTI group), including ameloblastoma, squamous cell carcinoma and osteosarcoma of the jaw; *** P value less than 0.001.

Abbreviation: SCC, squamous cell carcinoma.

Supplementary Table 2 Surgical options of patients in LRTI group.

Surgical options	LRTI group n=60	Non-LRTI group n=175	P value
**CLND**			< 0.001***
No	7 (11.67%)	26 (14.85%)	
Unilateral	33 (55.00%)	137 (78.29%)	
Bilateral	20 (33.33%)	12 (6.86%)	
**Mandible**			0.961
Retain	24 (40.00%)	72 (41.14%)	
PI	17 (28.33%)	31 (17.71%)	
US	9 (15.00%)	59 (33.71%)	
BS	10 (16.67%)	13 (7.43%)	
**Maxilla**			0.102
Retain	58 (96.66%)	157 (89.71%)	
UPCR	1 (1.67%)	15 (8.57%)	
BPCR	1 (1.67%)	3 (1.71%)	
**Masticatory muscle**			0.905
Retain	43 (71.67%)	124 (70.86%)	
Defect	17 (28.33%)	51 (29.14%)	
**Flap type**			0.010 *
ALTF	31 (51.67%)	64 (36.57%)	
FOMF	15 (25.00%)	37 (21.14%)	
Others	14 (23.33%)	74 (42.29%)	

Note: * P value less than 0.05.*** P value less than 0.001.

Abbreviation: CLND, cervical lymph node dissection; PI, paramedian incision; US, unilateral segmentectomy; BS, bilateral segmentectomy; UPCR, unilateral partial or complete resection; BPCR, bilateral partial or complete resection; ALTF, anterolateral thigh flap; FOMF, fibula osseo-myocutaneous flap.

Supplementary Table 3 Etiological test results in LRTI group patients.

	**Cases**	**Ratio**
**Bacterial culture**		
Klebsiella pneumoniae	8	18.18%
Pseudomonas aeruginosa	7	15.91%
Acinetobacter baumannii	6	13.64%
Staphylococcus aureus	5	11.36%
Streptococcus pneumoniae	3	6.82%
Others	15	34.09%
**Drug resistance**		
No drug resistance	13	35.13%
MDR	17	45.95%
Non-MDR	7	18.92%

Abbreviation: MDR, multi-drug resistance.

Supplementary Table 4 The multicollinearity test for multivariate analysis.

Characteristics	LRTI	non-LRTI	Multivariate analysis	
	(n=60)	(n=175)	Tolerance	VIF
**PCT** [a]	0.79±1.58	0.22±0.17	0.824	1.214
**CRP** [a]	92.18±53.39	30.90±28.46	0.735	1.361
**Tumor T stage**			0.890	1.123
≤2	4 (7.69%)	49 (35.77%)		
3	20 (38.46%)	48 (35.04%)		
4	28 (53.85)	40 (29.20%)		
**SSAV**				
Range [a]	32.28±22.02	20.48±15.61	0.171	5.823
Increase [a]	23.47±19.24	8.16±13.61	0.457	2.189
Decrease [a]	26.02±18.58	12.67±11.94	0.220	4.539

Note: [a] mean ± standard deviation.

Abbreviation: VIF, variance inflation factor; PCT, procalcitonin; CRP, C-reactive protein; SSAV, Subglottic sputum aspiration volume.

Supplementary Table 5 Patients' demographic characteristics in the validation cohort.

	Validation cohort (n=66)
**Gender**	
Male	51 (77.27%)
Female	15 (22.73%)
**Age(year)** [a]	53 ± 11
**Smoking history**	
Yes	32 (48.48%)
No	34 (51.52%)
**Alcohol consumption**	
Yes	17 (25.76%)
No	49 (74.24%)
**Chewing betel nut**	
Yes	7 (10.61%)
No	59 (89.39%)
**Diabetes**	
Yes	8 (12.12%)
No	58 (87.88%)
**Pre-operative HNSH**	
Yes	11 (16.67%)
No	55 (83.33%)
**Pre-operative RTH**	
Yes	4 (6.06%)
No	62 (93.94%)
**Pre-operative CTH**	
Yes	3 (4.55%)
No	63 (95.45%)
**LRTI**	
Yes	11 (16.67%)
No	55 (83.33%)
**Days of intubation** [a]	4.36 ± 1.31

Note: [a] mean ± standard deviation.

Abbreviation: HNSH, head and neck surgery history; RTH, radiotherapy history; CTH, chemotherapy history.

2 Supplementary Figures

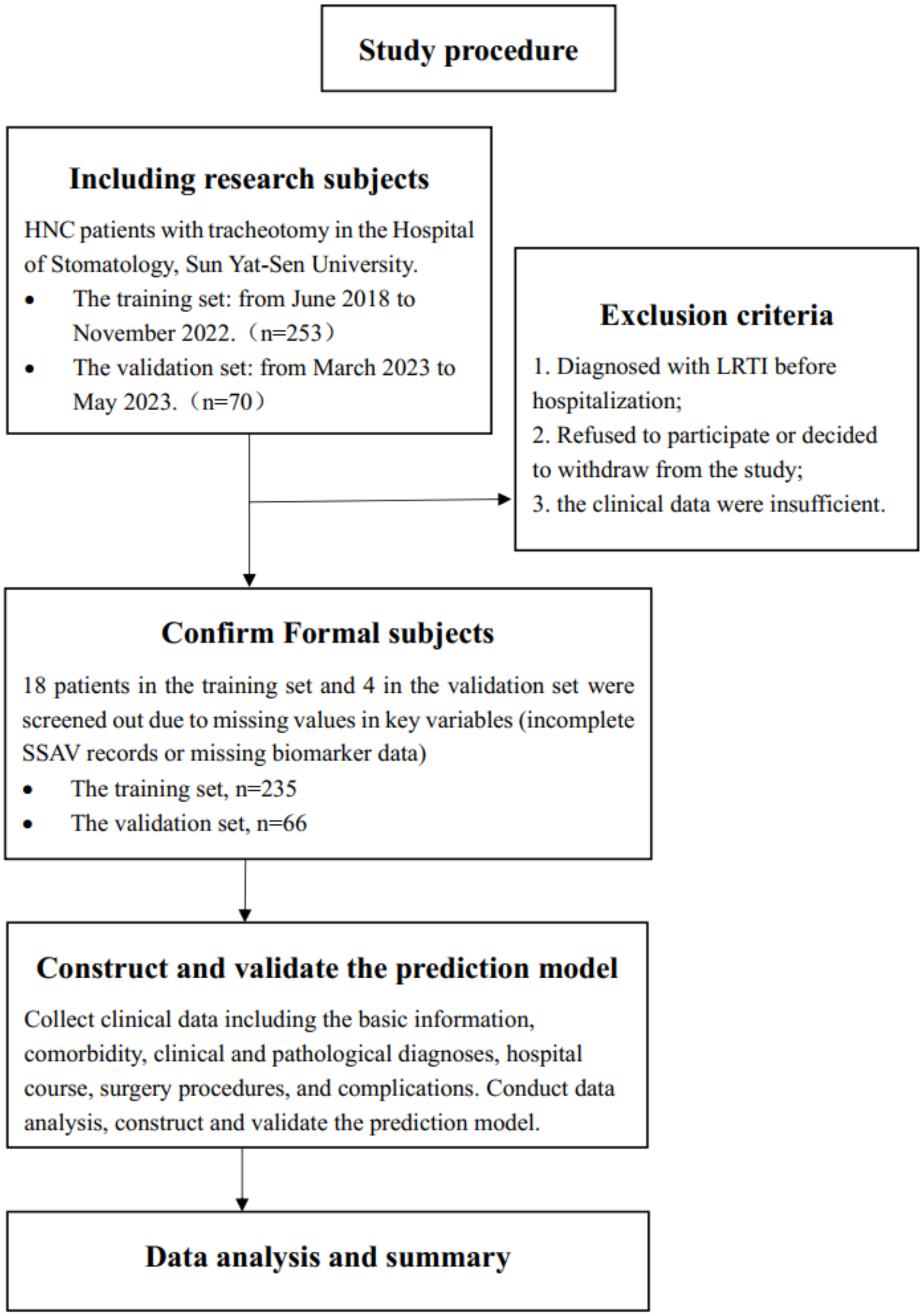

Supplementary Figure 1. Flowchart of the patient recruitment and selection process. The study comprised two phases: the retrospective training phase from June 2018 to November 2022 and the validation phase from March 2023 to May 2023. A total of 235 patients were included in the training set for model development, and 66 patients were included in the validation set. Abbreviations: HNC, head and neck cancer; LRTI, lower respiratory tract infection.

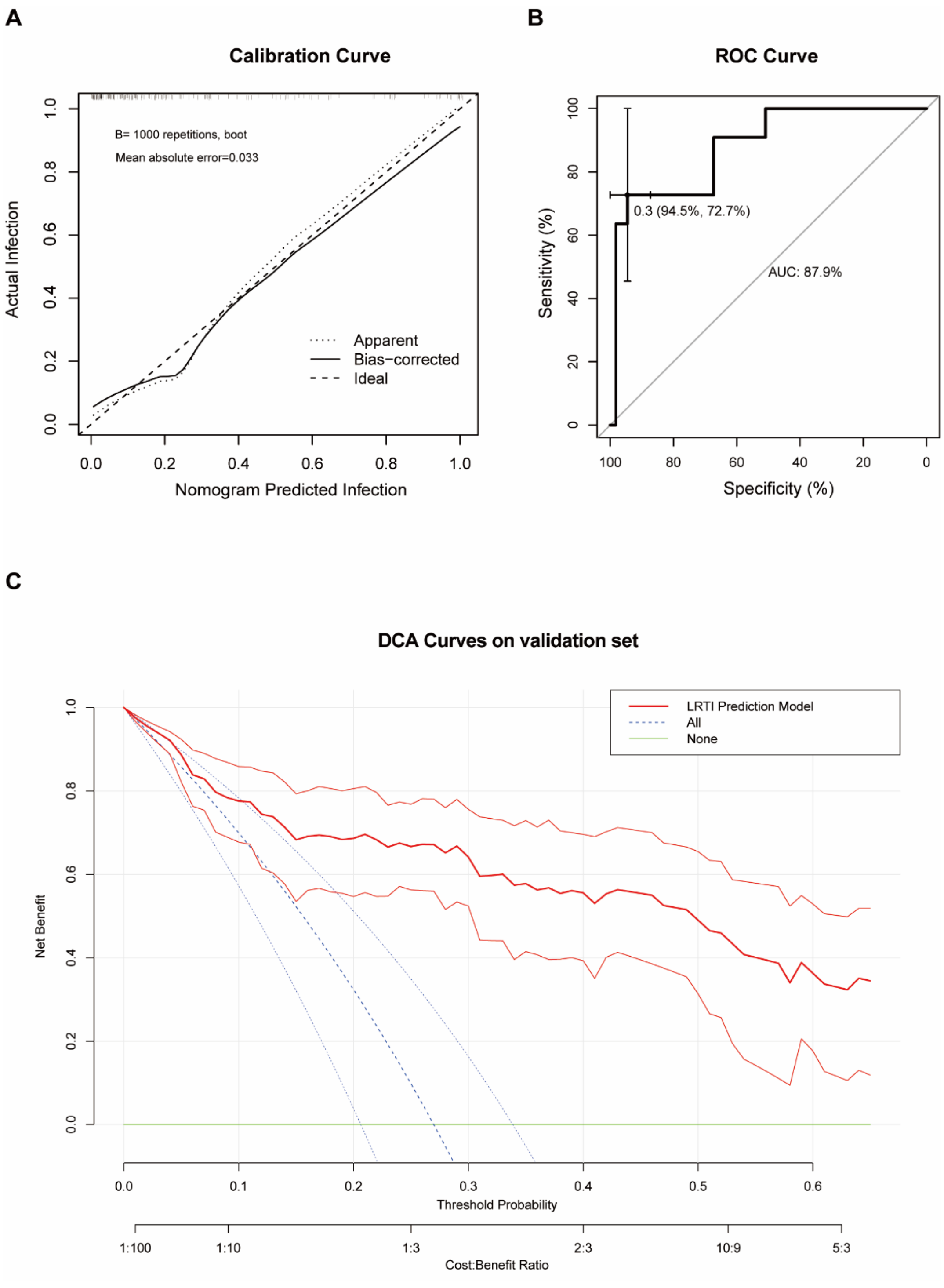

Supplementary Figure 2. Evaluation of the predictive model. (A, B) Calibration curve of the nomogram and Receiver Operating Characteristics (ROC) curve in the validation set. AUC: area under the curve. (C) Decision curve analysis (DCA) of the prediction model for postoperative LRTI on validation set. The y-axis measures the net benefit, and the x-axis measures the threshold probability. The red solid line represents the decision curve of the constructed nomogram. The gray dashed line represents the "treat-all" strategy (presented as All), and the horizontal green line represents the "treat-none" strategy (presented as None).
